# The Effect of Enteral Tube Feeding on Patients’ Health-Related Quality of Life: A Systematic Review

**DOI:** 10.3390/nu11051046

**Published:** 2019-05-10

**Authors:** Omorogieva Ojo, Edel Keaveney, Xiao-Hua Wang, Ping Feng

**Affiliations:** 1 Faculty of Education and Health, University of Greenwich, London SE9 2UG, UK; 2 Rockfield Medical Devices, Galway H91 DCH9, Ireland; edel@rockfieldmd.com; 3 The School of Nursing, Soochow University, Suzhou 215006, China; wangxiaohua@suda.edu.cn (X.-H.W.); fengping@suda.edu.cn (P.F.)

**Keywords:** enteral nutrition, enteral tube feeding, Quality of life, QoL, home enteral nutrition, enteral feed, patients, systematic review

## Abstract

Patients with functional gastrointestinal tract who are unable to meet their nutritional requirements may benefit from the use of enteral nutrition via feeding tubes which could be nasogastric, percutaneous endoscopic gastrostomy and jejunostomy. Although enteral tube feeding has been shown to promote nutritional status, improve wound healing, and enhance patients’ quality of life (QoL), evidence of tube and feed complications and reduced QoL has also been reported. Despite the increasing prevalence of patients on enteral tube feeding, no systematic review examining the role of enteral tube feeding on patients’ QoL appears to have been published. Aim: The aim of this systematic review is to evaluate the effect of enteral tube feeding on patients’ QoL. Method: Three databases (EMBASE, Pubmed, and PsycINFO) plus Google Scholar were searched for relevant articles based on the Population, Intervention, Comparator, Outcomes (PICO) framework. The review was in line with preferred reporting items for systematic reviews and meta-analyses (PRISMA) guidelines and involved the use of synonyms and medical subject headings. In addition, search terms were combined using Boolean operators (AND/OR) and all the articles retrieved were exported to EndNote for de-duplication. Results: Fourteen articles which met the criteria were included and three distinct areas were identified: the effect of early versus late enteral tube feeding on QoL; the QoL of patients on gastrostomy versus standard care, and the effect of enteral tube feeding on QoL. Overall, nine studies reported improvement in the QoL of patients on enteral tube feeding, while five studies demonstrated either no significant difference or reduction in QoL. Some factors which may have influenced these outcomes are differences in types of gastrostomy tubes, enteral feeding methods (including time patients spent connected to enteral feed/pump), and patients’ medical conditions, as well as the generic and/or type of QoL measuring instrument used. Conclusion: Most reviewed studies suggest that enteral tube feeding is effective in improving patients’ QoL. The use of enteral tube feeding-specific QoL measuring instruments is recommended for future research, and improved management strategies including use of mobile enteral feeding pumps should further enhance patients’ QoL. More studies on the effect of delivery systems/enteral feeding pumps on QoL are needed as research in this area is limited.

## 1. Introduction

There is evidence of increasing prevalence of patients on enteral tube feeding in the UK and around the world [1,2] and this calls for greater scrutiny in terms of evaluating the impact of this method of feeding on patients’ quality of life (QoL). In patients with neurological conditions such as stroke, swallowing problems and undernutrition are common and nutritional status can deteriorate in various clinical settings and this has been linked to increased fatality and poor functional status [3,4,5]. Therefore, enteral tube feeding is an effective method of providing nutritional support to these patients and other patients with functional guts who are unable to meet their nutritional requirements through the oral route alone due to a range of conditions [6,7]. Therefore, patients with chronic conditions such as stroke, multiple sclerosis, moto—neuron disease and dementia which may impact the patient’s swallowing ability usually require enteral nutrition support to promote clinical outcomes [5,8]. Enteral tube feeding may also be useful in patients with obstructive pathology of the oropharynx such as head and neck cancer patients either as prophylactic measures or as post-radiotherapy interventions [9] due to the effect of radiation on swallowing reflexes/muscle and radiation induced mucositis [10].

Sometimes, enteral tube feeding is needed to support patients with human immuno-deficiency virus (HIV), those who fail to thrive and individuals with learning and intellectual disability and may involve patients in their own homes, residential care, nursing homes, acute hospitals including intensive care units [6,11,12]. There is evidence that enteral tube feeding can improve wound healing, reduce length of hospital stay, prolong life and relatively save costs [11]. For instance, diabetes specific enteral formula has been found to be effective in managing patients with diabetes on enteral nutrition [13] while in patients with esophageal cancer, early enteral nutrition was effective in reducing the incidence of postoperative pulmonary infection, enhancing early recovery and reducing length of hospital stay and hospital cost [14]. Enteral tube feeding has also shown promising results in the management of Crohn’s disease as it provided equal or higher remission rates than the current medication in use [15]. However, despite the merits in the use of enteral tube feeding, challenges such as its impact on patients’ QoL remain.

The nature of enteral feeding systems may be implicated in these problems. These include the feed and enteral feeding tube which is usually placed or inserted in the patient [5]. Other enteral feeding equipment and accessories including the feeding pump, the drip stand and syringes are essential features of enteral nutrition provision which can bring challenges and potentially impact on patients’ QoL.

QoL has been defined as the way in which illness, pain, reduced motor activity and unease may influence daily behavior, social activities, psychological well–being and other aspects of an individual’s life [16]. Therefore, when evaluating QoL, four dimensions are usually considered including motor activity, functional, psychological and social dimensions [16]. In particular, QoL provides a measure of general wellbeing, including both positive and negative features of life [17]. A range of QoL measurement tools such as the EuroQoL-5-Dimensions (EQ-5D) which consists of questionnaires and Visual Analogue Scale (VAS) [16,18,19], World Health Organization (WHO) [20], and the more specific enteral nutrition NutriQoL [21] are now available. Govindaraju et al. [17] in their systematic review of dietary patterns and QoL in older adults also selected articles employing a range of generic QoL measuring tools. The authors noted that QoL is both subjective and objective constructs and measures the subjective of health against the objective assessments of functioning and/or health status [17].

It remains unclear whether these QoL measuring instruments can identify the effect of the various enteral feeding tubes such as nasogastric (NGT), nasojejunal (NJ), percutaneous endoscopic gastrostomy (PEG), radiologically inserted gastrostomy (RIG) and percutaneous endoscopic jejunostomy (PEJ) which require different procedures for placement in the respective anatomical sites on QoL [5,6,21]. In addition, the care and management of these tubes are also different. The challenges of enteral tube feeding such as tube blockage, kinking and leakage, stoma site infection, overgranulation of stoma site and buried bumper syndrome may also have effect on patients’ QoL [22].

Other potential problems include the enteral feeding pump that delivers the feed and to which patients could be connected for many hours [2,22]. In addition, continuous enteral feeding method can restrict patients’ mobility and the noise from the pump can cause sleep disturbance [22]. However, studies on patients and caregivers’ experiences of enteral feeding pumps and the impact on QoL appear limited [23]. Patients on enteral tube feeding are sometimes unable to tolerate the feed and may suffer from bloating, diarrhea, constipation, nausea and vomiting [22,24]. Enteral tube feeding can also have significant impact on body image [22]. In these circumstances, the patients’ QoL may be affected.

The approaches to enteral nutrition provisions in hospitals and in community settings may also affect patients’ QoL as there are variations in the provision of enteral tube feeding in the UK and globally [1,6]. For example, in the UK, there are established home enteral nutrition (HEN) teams in some commissioning groups while there are none in others [1].

Given the role of enteral tube feeding in terms of its importance in prolonging life and reducing length of hospital stay in patients with different health conditions and the range of challenges that have been highlighted, it is not surprising that while some studies have noted the merits of enteral nutrition provisions, other articles have drawn our attention to its demerits and the negative effects on patients’ QoL. However, despite the studies conducted in this area, it would appear that there is no systematic review that has attempted to explore the role of enteral tube feeding on patients’ QoL.

Aim: The aim of this systematic review is to evaluate the effect of enteral tube feeding on patients’ QoL.

Research question: Do enteral tube feeding provisions impact on patients’ QoL?

## 2. Method

This systematic review has been conducted in line with the preferred reporting items for systematic reviews and meta-analyses (PRISMA) [25].

### 2.1. Types of Studies

Due to the nature of the area being reviewed (enteral tube feeding and QoL), a range of study designs including randomized controlled trials, cross-sectional studies, prospective cohort studies, uncontrolled clinical trial and retrospective reviews were included in this review.

### 2.2. Participants and Interventions

Participants were patients on enteral tube feeding involving different enteral feeding tubes and various types of enteral feed. The studies included evaluated the effect of various gastrostomy tube placements, the timing of tube placement, HEN, and compared enteral feed and standard care on QoL.

### 2.3. Outcome Measures

The outcome of interest was the QoL of patients based on the use of different QoL measuring scales. The tools used in the studies selected included the EuroQoL 5D (EQ5D) index and EQ5D VAS [26], Short-Form 36 (SF-36) with social, physical, psychological and occupational domains of QoL [27], European Organization for Research and Treatment of Cancer (EORTC) quality of life questionnaire (QLQ-C30) and QLQ-OES19 (esophageal cancer specific); Inflammatory Bowel Disease Questionnaire (IBDQ).

### 2.4. Search Terms and Search Strategy

**Figure 1:**
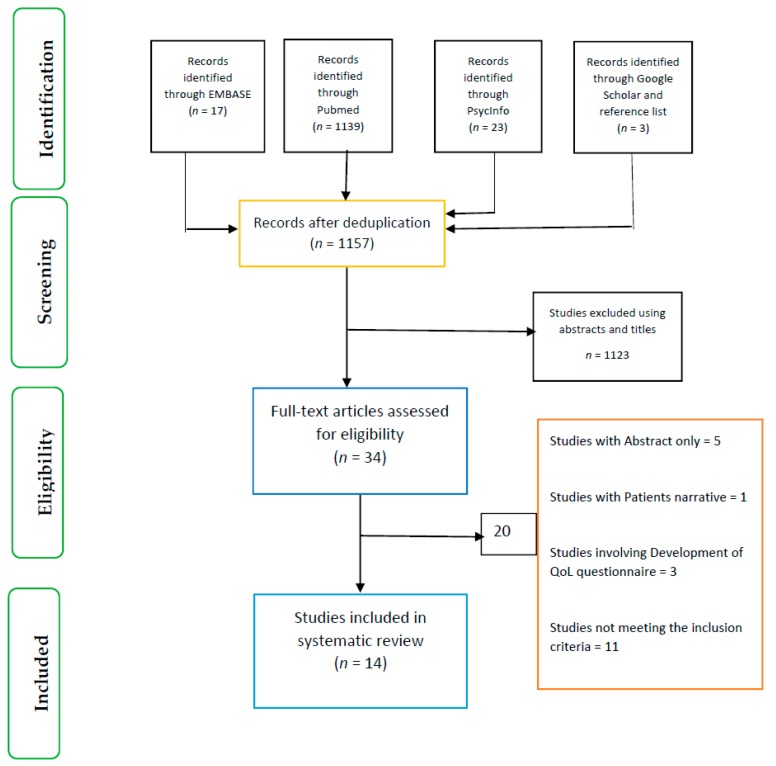
Prisma flow chart.

The search strategy for this review was based on the Population, Intervention, Comparator, Outcomes (PICO) framework (Table 1). The search terms are outlined in Table 1 and involved the use of synonyms and medical subject headings (Mesh) and the combination of the search terms using Boolean operators (AND/OR). Three databases (EMBASE, Pubmed and PsycINFO) plus Google Scholar were searched for relevant articles (Figure 1). The reference list of articles was also searched for articles of interest. The searches were conducted by one researcher (O.O.) and cross checked by three other researchers (E.K., X.-H.W. and P.F.). All the articles retrieved from the databases were first exported to EndNote (Analytics, Philadelphia, PA, USA) for de-duplication.

### 2.5. Inclusion and Exclusion Criteria

Searches were conducted in the three databases from the date of inception to 27 December 2018. The following were the inclusion criteria: studies involving patients on only enteral tube feeding irrespective of the medical condition and type of enteral feeding tube; studies involving patients older than 18 years; studies involving patients’ QoL.

The exclusion criteria were: studies involving patients on home parenteral nutrition; patients on both home parenteral and enteral tube feeding; patients on oral nutritional supplements; studies involving children aged below 18 years; studies involving family members and/or healthcare professionals; letters; studies comparing different feeding tubes; studies based only on medical conditions and gender of patients; studies with abstract only and insufficient information/data.

### 2.6. Quality Assessment

The quality of each study included in this review was evaluated by using the critical appraisal skills program (CASP) [28] tool.

### 2.7. Data Extraction

Data was extracted from the studies selected by one researcher (O.O.) and cross checked by three other researchers (E.K., X.-H.W. and P.F.) (Table 2).

## 3. Results

Three studies each were conducted in the United Kingdom, France and China. Furthermore, Ireland, Australia, Iran, Germany and New Zealand each had one study (Table 2). These studies were a mix of randomized controlled trials [26,33], prospective cohort studies [27,31,34,37], non-randomized studies [39,40], cross-sectional studies [30,32,38], retrospective review [29], cross-sectional and longitudinal studies [36] and uncontrolled clinical pilot study [35].

Based on the objectives, the interventions and outcomes of the studies, the following three areas were identified from the review;

- The effect of early versus late enteral tube placement/feeding on QoL
- QoL of patients on gastrostomy compared with standard care
- The effect of enteral tube feeding on QoL

### 3.1. The Effect of Early Versus Late Enteral Tube Placement/Feeding on QoL

Two studies [26,29] evaluated the effect of early versus late enteral tube placement/feeding or standard care on QoL. Baker et al. [26] found that early enteral feeding (intraoperative nasojejunal tube placement) for malnourished women with advanced epithelial ovarian cancer did not significantly improve patient’s QoL 6 weeks postoperatively compared to standard care, but may improve nutritional status. This study was based on the use of nasojejunal tube and EQ5D Index tool and VAS. On the other hand, Morton et al. [29] noted that early PEG insertion and shorter PEG duration are associated with more favorable QoL-related outcomes based on the University of Washington Head and Neck Disease-Specific Measure (UW-QoL).

### 3.2. Quality of Life of Patients on Gastrostomy Compared with Standard Care

Five studies [27,30,31,32,33] explored the effect of different gastrostomies on QoL. Kurien et al. [31] observed no significant longitudinal changes in mean EuroQoL index scores for patients (0.70 before vs. 0.710 3 months after; *p* = 0.83) following gastrostomy insertion. In fact, Rogers et al. (2007) reported that patients with PEGs (at 34 months) reported significant deficits in all UW-QoL domains compared to non-PEG or PEG-removed (at 7 months) patients and reported a much poorer QoL.

Bannerman et al. [27] found no significant difference (*p* > 0.05) in SF-36 scores at the time of tube placement and 1, 6, 12 months follow-up, except for physical function score. However, the PEG-Qu assessment showed at 6 and 12 months, 71% and 75% of patients respectively expressed a positive overall effect of gastrostomy on their QoL. In addition, Hossein et al. [30] demonstrated significant improvement (*p* < 0.005) 6 months after PEG placement in the QoL index scores. There is also evidence that prophylactic gastrostomy can improve post-treatment QoL for unresectable head and neck cancer patients, after adjusting for other potential predictive QoL factors [33] based on the SF36 and QLQ-C30 scores.

### 3.3. The Effect of Enteral Tube Feeding on Quality of Life

Seven studies [34,35,36,37,38,39,40] assessed the effect of enteral tube feeding on QoL. Based on the QLQ-C30 and QLQ-OES19 tools, Donohue et al. [34] found that weight loss and negative consequences on QoL occurred in most post-esophageal cancer surgery patients, despite supplemental HEN for further 4 weeks. These findings were similar to that of Loeser et al. [36] who noted that QoL is reduced in patients on home enteral tube feeding (HETF), although HETF can prevent further weight loss and improve some aspects of QoL within 4 months. This study relied on the QLQ-C30 tool.

Schneider et al. [38] reported that although QoL is poor in patients on HEN (for 25 months) compared to age and sex matched general population, most patients described an improvement in their QoL following the initiation of HEN using the SF36 tool, EQ5D Index and VAS. Similarly, Roberge et al. [37] found that in patients treated for head and neck or esophageal cancer on HETF, overall, QoL slightly improved 3 weeks post-discharge, based on the QLQ-C30 scale. Furthermore, Wu et al. [39] observed that minimally invasive esophagectomy and subsequent treatment with 3 months HEN can improve the QoL and reduce the risk of malnutrition in preoperatively malnourished patients based on the QLQ-C30 and PG-SGA. In the Zeng et al. [40] study, the authors used the combined QLQ-C30 and QLQ-ES18 to demonstrate that compared to the control group, the HEN group achieved higher Global QoL scores, and most of their functional index scores were better, at 4 and 12 weeks after surgery. However, 24 weeks after surgery, QoL indexes did not differ significantly between the two groups. A 4-week treatment based on exclusive enteral nutrition (EEN) also improved QoL significantly in adults with active Crohn’s disease using the IBDQ [35].

## 4. Discussion

Evaluation of the selected studies on the impact of enteral tube feeding on QoL resulted in the emergence of 3 distinct areas (the effect of early versus late enteral tube feeding; the QoL of patients on gastrostomy versus standard care, and the effect of enteral tube feeding on QoL) with different outcomes observed in each area. Overall, nine [27,29,30,33,35,37,38,39,40] of the 14 studies included showed improvement in the QoL of patients on enteral tube feeding, while the remaining five studies [26,31,32,34,36] demonstrated either no significant difference or reductions in QoL (Table 2). Gastrostomies resulted in improved QoL compared to standard care in the majority of studies across a variety of patient conditions over a 6–12 month post gastrostomy timeframe [27,30,33], although in the other studies [31,32], there was either no significant change or a decrease in QoL over 3–34 months post gastrostomy tube placement. Similarly, in the majority of studies, enteral tube feeding /HEN showed a positive effect on QoL in a range of patient conditions, over a 3 week–25 month timeframe [35,37,38,39,40], while in the other studies [34,36], enteral tube feeding /HEN showed a reduction in QoL over a 4 to 6 month period.

The differences observed in the outcomes of the studies across the three areas outlined above could be due to a range of factors. These possible factors and the implications for research and practice will be discussed.

### 4.1. Factors Influencing the Role of Enteral Tube Feeding on Patients’ QoL

These factors may include the types of gastrostomy feeding tubes, the various chronic conditions requiring enteral tube feeding, the enteral feeding methods, the time spent by patients being connected to enteral feed/pump and the different clinical settings [2,22,27,38]. Furthermore, the use of generic QoL measurement tools is another factor which may influence the outcome of studies on the role of enteral tube feeding on QoL. In this review, different QoL measurement tools were used including the EQ5D with the EQ5D VAS [26,31], the SF-36 and the PEG Qu which has 10 questions, specific about gastrostomy tube and QoL [27]. The vast array of generic QoL measurement tools used in the studies selected were developed and validated by different researchers and in different population group, which may partly explain the differences in the outcomes of the studies [21]. Govindaraju et al. [17] in a previous review noted that it is possible for two individuals with identical health status to have different QoL based on their expectation and their capacity in health or illness, socio-economic status, age and social support.

There were a range of enteral feeding tubes such as NGT, NJ, PEG and RIG, used by the patients in the selected studies. These gastrostomy tubes vary in their indications, method of tube placement, anatomical sites, complications, and care and management [41,42,43]. Therefore, the impact of the different gastrostomy tubes on QoL will be different.

Similarly, the pathophysiology of the long-term conditions requiring enteral tube feeding such as stroke, Crohn’s disease and cancers are different [43,44], and the differing treatment options may also have implications for patients’ QoL. The settings where the studies included took place varied from the community such as HEN to acute hospitals [2,43,45] and these different settings may also influence the impact of enteral tube feeding on patients’ QoL. Methods of enteral feeding could be in the form of bolus, gravity and the use of stationary or mobile enteral feeding pumps and each of these enteral feeding methods have their advantages and drawbacks in terms of how they impact patients’ QoL [46]. For example, the long hours spent by patients who are connected to enteral feed/pump may affect family life, social activities and QoL [47] and the use of mobile pumps and carry bags are possible strategies for ameliorating these challenges.

The differences observed with respect to the impact of enteral tube feeding on QoL is underscored by the fact that although this method of feeding has significant advantages, it is not perfect [48]. There is evidence that this feed delivery route is efficacious, lowers costs and safety compared with parenteral nutrition [6]. However, there are also physical complications, poor psychological outcomes such as depression associated with patients on enteral tube feeding [6]. Fears of being dependent and institutionalized, and the inability to perform activities of daily living are some of the challenges of enteral tube feeding which may impact patients’ QoL [6]. Other potential problems associated with enteral tube feeding are interference with family life, intimate relationships, social activities and hobbies [32]. Despite the difficulties of enteral tube feeding highlighted above, gastrostomy tube placement is based on the understanding that enteral tube feeding provides more clinical benefits, patient comfort, functional status and QoL when compared to malnutrition [6].

### 4.2. Implications for Research and Practice

It would appear that some of the studies included in this review recognized the limitations of using generic QoL measurement tools for evaluating patients on enteral tube feeding by including PEG specific questionnaires to complement these tools. For example, Rogers et al. [32] used the UW—QoL and PEG questionnaire for their study. In addition, the SF-36 and the PEG Qu which has 10 questions, specifically about gastrostomy tube and QoL were used by Bannerman et al. [27] to explore QoL in patients on enteral tube feeding. Cuerda et al. [21] went further to develop and validate a specific questionnaire (NutriQoL) to assess health-related QoL in patients on HEN. One of the justifications for their study was that several studies on patients receiving HEN used generic measurement instruments to assess patients’ QoL. The authors noted that these generic tools were not sensitive enough in identifying the effect of enteral tube feeding on patients’ QoL. Based on the process used in developing and validating the NutriQoL questionnaire, it was concluded that this measurement tool is valid, reliable and useful instrument for assessing the QoL of patients on HEN irrespective of the disease and/or the route of administration [46,49].

Strategies for ameliorating some of the challenges of enteral tube feeding and improving patients’ QoL should be promoted. These approaches could involve the development of technology such as improved mobile enteral feeding pump with less noise, development of HEN services in the community to provide specialist enteral nutrition services and support patients to reduce the risk of feed, tube and pump complications and thus improve their QoL.

## 5. Limitations of the Review

The use of generic QoL tools/questionnaires to evaluate the impact of enteral tube feeding on patients’ QoL presents a significant limitation. However, this has been discussed extensively in this review to raise awareness among researchers and recommendations for future research have been suggested.

## 6. Conclusions

Most studies in this review suggest that enteral tube feeding is effective in improving patients’ QoL. The varying outcomes of the effect of enteral tube feeding on QoL across the three areas may be partly explained by differences in types of gastrostomy tubes, enteral feeding methods (including time patients spent connected to enteral feed/pump), and patients’ medical conditions, as well as the type of QoL measuring instrument used.

However, the use of an enteral nutrition specific QoL measuring tool which does not discriminate in terms of the type of enteral feeding tube and patients’ condition and has been validated is recommended for evaluating the impact of enteral tube feeding on patients’ QoL. In addition, improved management strategies including the use of mobile enteral feeding pumps should further enhance patients’ QoL. More studies on the effect of delivery systems/enteral feeding pumps on QoL are needed as research in this area is limited.

## Figures and Tables

**Table 1:** Search Terms and Search Strategy.

Patient/Population	Intervention	Comparator	Outcomes of Interest	Combining Search Terms
Patients on enteral tube feeding	Enteral nutrition	Control	Quality of life	
Patients on enteral tube feeding OR Enteral feeding OR Enteral nutrition OR Feeding, enteral OR Nutrition, enteral	Nutrition, Enteral OR Enteral feeding OR Feeding, Enteral OR Tube feeding OR Feeding, Tube OR Gastric feeding tubes OR Feeding tube, Gastric OR Feeding tubes, Gastric OR Gastric feeding tube OR Tube, Gastric feeding OR Tubes, Gastric feeding	Control OR Standard diet OR Normal diet as tolerated OR Baseline values	Life quality OR Health-related quality of life OR Health-related quality of life OR HRQoL OR QoL	Column 1 AND Column 2 AND Column 3

**Table 2:** Characteristics of the articles included in this review (N = 14).

Study Reference	Country of Study	Study Type/Design	Sample Size	Age (Years)	Aim/Objective	Interventions Including Type of Tube and/or Enteral Feeding	Results of QoL Scores Following Interventions	Conclusion
The effect of early versus late enteral tube placement/feeding on QoL								
Baker et al. (2015) [26]	Australia	Phase III multicenter, randomized clinical trial	Intervention N = 53 Standard care N = 56 Total N = 109	Mean (SD) Intervention: 61.8 (11.4) Standard care: 63.7 (12.7)	Whether early postoperative enteral nutrition for malnourished women with advanced epithelial ovarian cancer can improve their QoL compared to Standard care	Nasojejunal tube: Early enteral feeding versus Standard care	**Baseline EQ5D Index (SD)** Intervention = 0.70 (0.20) Standard care = 0.65 (0.22) **6 weeks Postoperatively EQ5D Index:** Intervention = 0.78 (0.22) Standard care = 0.76 (0.15) **30 days Post-chemotherapy EQ5D Index** Intervention = 0.85 (0.13) Standard care = 0.78 (0.16) **Baseline VAS (SD)** Intervention = 60 (23) Standard care = 51 (20) **6 weeks Postoperatively VAS (SD)** Intervention = 69 (20) Standard care = 61 (21) **30 days Post-chemotherapy VAS** Intervention = 72.8 (15.2) Standard care = 65.2 (19.2)	Early enteral feeding did not significantly improve patient’s QoL compared to standard care but may improve nutritional status
Morton et al. (2009) [29]	New Zealand	Retrospective chart review over a 24-month period.	N = 36	Median = 52	To examine the factors associated with PEG insertion and the effects of PEG use on QoL and functional outcomes in head and neck cancer (HNC) patients receiving chemoradiotherapy	PEG insertion: (1) tube inserted before treatment or within 1 month of commencement of treatment (2) tube inserted 1 month or more after start of treatment	Patients who still had PEG in situ at the time of the survey had a significantly worse total QoL score (*p* = 0.006) Overall QoL Score: Nutrition mode at time of study = 0.363 (*p* = 0.063) PEG in situ at time of study = 0.518 (*p* = 0.006) Longer PEG duration predicted poor overall QoL (*p* < 0.01)	Early PEG insertion and shorter PEG duration are associated with more favorable QoL-related outcomes
Quality of Life of patients on gastrostomy compared with standard care								
Bannerman et al. (2000) [27]	United Kingdom	Cross-sectional and prospective cohorts	Prospective study: N = 54	Median = 58	To determine the impact of gastrostomy on QoL	Patients were assessed prior to gastrostomy (endoscopic or radiological) placement at baseline, 1, 6 and 12 months	No significant difference in SF-36 scores at the time of tube placement and 1, 6, 12 months follow-up (*p* > 0.05), except for physical function score (Mean ± SD) **Baseline scores** = 43.8 (34.9) **6 months** = 14.7 (20.9) *p* = 0.01. No significant difference in the proportion of patients showing that gastronomy had a positive impact on their QoL (*p* > 0.05). **Based on the PEG-Qu assessment, at 6 and 12 months**: 71% and 75% of patients respectively expressed a positive overall effect on their QoL	Most patients can cope adequately with the care of gastrostomy, despite considerable impairment of physical function. QoL of patients fed via gastrostomy is independent of nutritional outcome. Overall, the positive impact of gastrostomy on QoL was perceived in 55% of patients and 80% carers
Hossein et al. (2011) [30]	Iran	Cross-sectional study	N = 100	Mean (SD) 59.73 ± 18.16	To assess the perspectives of patients regarding the acceptability of PEG tube placement and evaluate the outcomes	PEG tube	**QoL index scores (Mean)** Pre-PEG: 19.25 ± 11.85 6 months after: 32.08 ± 27.74 When comparing the mean QoL index scores before and after PEG placement there was significant improvement (*p* < 0.005) after PEG placement	PEG tube is a minimally invasive gastrostomy method with low morbidity and mortality rates, and is easy to follow-up and to replace when blockage occurs
Kurien et al. (2017) [31]	United Kingdom	Prospective multicenter cohort study	N = 100 (patients) N = 100 (caregivers) N = 200 (control)	Mean (SD) Patients: 67 (14.7) Caregivers: 65 (12.2) Control: 60 (10.1)	To determine how gastrostomies affect QoL in patients and caregivers	PEG (55%) + RIG (45%)	**Baseline (before gastrostomy) versus 3 months post insertion (Mean ± SD)** No significant longitudinal changes in mean EuroQoL index scores for patients (0.70 before vs. 0.710 after; *p* = 0.83) or caregivers (0.95 before vs. 0.95 after; *p* = 0.32) following gastrostomy insertion	QoL did not significantly improve after gastrostomy insertion for patients or caregivers. Gastrostomies may help maintain QoL
Rogers et al. (2007) [32]	United Kingdom	Cross-sectional survey	N = 243	Mean (SD) 65 (12)	To devise, pilot and survey a PEG specific questionnaire and relate outcomes to QoL	PEG	Global measures score (0–100) QoL (Mean; SE) as measured by UW-QoL **Never had PEG:** 63 (1) **PEG removed at 7 months:** 68 (3) **Still has PEG at 34 months:** 41 (4)	Patients with PEGs reported significant deficits in all UW-QOL domains compared to non-PEG or PEG-removed patients and reported a much poorer QoL
Salas et al. (2009) [33]	France	Randomized, controlled study	N = 39 No systematic gastrostomy (standard group) = 18 Systematic gastrostomy (experimental group) = 21	Mean (SD) Standard = 60.0 ± 4.5 Experimental = 58.7 ± 7.7	To assess the impact of prophylactic gastrostomy on the 6-month QoL, and to determine the factors related to this QoL	Systematic percutaneous gastrostomy versus no systematic gastrostomy	QoL at Inclusion SF36 Score Standard: 49.4 ± 25.1 Experiment: 59.2 ± 21.8 (*p* = 0.19) EORTC (QLQ-C30): Standard: 57.8 ± 25.8 Experiment: 63.0 ± 24.1 (*p* = 0.37) QoL at 6 months was significantly higher in the group receiving systematic prophylactic gastrostomy (*p* = 10^−3^)	Prophylactic gastrostomy improves post-treatment QoL for unresectable head and HNC, after adjusting for other potential predictive QoL factors
The effect of Enteral tube feeding on QoL								
Donohoe et al. (2017) [34]	Ireland	Prospective cohort study	N = 149	Mean (SD) 62 ± 9	To analyze the impact of supplemental HEN post-esophageal cancer surgery on quality of life	HEN	**QoL measured at baseline, preoperatively, and at 1, 3, and 6 months** Mean Global QoL decreased (*p* < 0.01) from 82 to 72. Global QoL (follow-up long-term) was not significantly different in those with <10% vs. >10% weight respectively (68.7 ± 20.6 vs. 70.95 ± 17.5, *p* = 0.519). With persistent weight loss (3–6 months postoperative, *n* = 12) there was clinically relevant decrease in QoL in physical (76.7 vs. 87.5, *p* = 0.066) and social function (76.4 vs. 87.8, *p* = 0.034)	Weight loss and negative consequences on QoL occurs despite supplemental enteral nutrition in majority of patients
Guo et al. (2013) [35]	China	Uncontrolled pilot clinical trial	N = 13	Mean (SD) 26.1 (3.8)	To determine the effect of exclusive enteral nutrition (EEN) on patients QoL in adults with active Crohn’s disease	Enteral nutrition	There were significant improvements in total IBDQ scores after 4-week EEN treatment (Mean ± SD) 128.3 ± 15.8 to 182.9 ± 24.2 (*p* < 0.001)	A 4-week treatment of EEN improves QoL significantly in adults with active Crohn’s disease and was acceptable by most patients
Loeser et al. (2003) [36]	Germany	Prospective cross-sectional (Study 1) Prospective longitudinal (Study 2: follow-up 4 months)	Cross-sectional N = 155 Longitudinal N = 56	Mean (SD) 64.3 ± 13.1	To assess QoL in patients on HETF.	HETF HETF/PEG insertion	Study 1: When compared with EORTC reference data, functional scales were lower in HETF patients and QoL was significantly lower in non-competent patients. Study 2: QLQ-C30 (N = 26) **PEG insertion:** 44.2 ± 19.7 **2 months:** 46.5 ± 16.0 **4 months:** 50.6 ± 1 Lower QoL was observed in non-competent than in competent patients	QoL is decreased in patients on HETF. Part of this explained by malnutrition. HETF can prevent further weight loss and improve some aspects of QoL
Roberge et al. (2000) [37]	France	Prospective study	N = 39	Mean = 58	To evaluate the impact of HETF on QoL in patients treated for head and neck or esophageal cancer. Evaluations were carried out 1st week and 3rd week post hospital discharge	HETF/PEG insertion	**QLQ-C30 Mean (SD)** Global health status: 45(19). Overall, QoL slightly improved 3 weeks post-discharge; some symptoms significantly improved (*p* < 0.05): constipation, coughing, social functioning and body image/sexuality	Home enteral tube feeding is a physically well accepted technique although some of the patients may experience psychosocial distress
Schneider et al. (2000) [38]	France	Cross-sectional study	N = 38	Mean (SEM) 56 ± 5	To assess both the QoL of long-term patients on HEN (for 25 ± 5 months) and the evolution of QoL after initiation of HEN	HEN vs. general population	**EQ-5D index** HEN: 0.54 ± 0.07 vs. General: 0.85 ± 0.0 (*p* < 0.05) Visual Analogue Scale HEN: 54.1 ± 4.2 vs. General: 82.5 ± 0.3 (*p* < 0.05) SF-36 (Mental Component Scale) HEN: 46.2 ± 2.6 vs. General: 51.8 ± 0.3 SF-36 (Physical Component Scale) HEN: 37.1 ± 2.1 vs. General: 46.5 ± 1.2 (*p* < 0.05)	QoL is poor in HEN patients compared to age and sex matched general population. Most patients describe an improvement in their QoL following the initiation of HEN
Wu et al. (2018) [39]	China	Single-center, prospective, non-randomized study	N = 142	Median (Range) Minimally invasive esophagectomy/laparoscopic jejunal feeding tube+HEN (MIE): 62 (45–80) Open esophagectomy/ nasojejunal feeding tube (OE): 61 (43–80)	To investigate the effect of 3 months HEN on QoL and nutritional status of esophageal cancer patients who were preoperatively malnourished.	MIE vs. OE	QoL (Global health status) (Mean ± SD) **Preoperative** MIE:69.9 (9.1) OE:70.1(10.3), *p* = 0.546 **2 weeks** MIE: 19.6 (7.5) OE: 18.4 (7.0), *p* = 0.821 **3 months** MIE: 55.7 (7.4) OE: 41.8 (7.0), *p* = 0.001	MIE and subsequent treatment with 3 months HEN can improve QoL and reduce the risk of malnutrition in preoperatively malnourished patients
Zeng et al. (2017) [40]	China	Non-Randomized Clinical trial	N = 60 HEN: N = 30 Control (Standard Care): N = 30	Mean (SD) HEN: 61.7 ± 8.4 Control: 59.3 ± 10.4	To characterize the effect of HEN on nutritional status and QoL of esophageal cancer patients who underwent Ivor Lewis esophagectomy for cancer	HEN vs. standard care	**Combined use of QLQ-C30 and QLQ-ES18** Compared to the control group, the HEN group achieved higher Global QoL scores, and most of their functional index scores were better. However, 24 weeks after surgery, QoL indexes did not differ significantly between the two groups	HEN can reduce the incidence of malnutrition or latent malnutrition and help restore QoL in the patients with esophageal cancer in the early period (24 weeks) after surgery

Abbreviations: EEN (Exclusive Enteral Nutrition); EQ5D Index (EuroQoL 5D) and EQ5D Visual Analogue Scale (VAS); SF-36 (Short-form 36); PEG (Percutaneous Endoscopic gastrostomy); PEG Qu (10 questions, specific about gastrostomy tube and QoL); EORTC (European Organization for Research and Treatment of Cancer) quality of life questionnaire (QLQ-C30) and QLQ-OES19 (esophageal cancer specific); HEN (Home Enteral Nutrition); HETF (Home Enteral Tube Feeding); HNC (Head and Neck Cancer); IBDQ (Inflammatory Bowel Disease Questionnaire); MIE (Minimally Invasive Esophagectomy); OE (Open Esophagectomy); PG-SGA (Patient Generated Subjective Global Assessment); QoL (Quality of Life); QLQ-ES18 (Esophageal module 18 questionnaire); RIG (Radiologically Inserted Gastrostomy); SD (Standard Deviation); SEM (Standard Error of Mean); UW-QoL (University of Washington Quality of Life questionnaire).
